# Artificial sweetener sucralose: a possible modulator of autoimmune diseases

**DOI:** 10.1038/s41392-023-01607-0

**Published:** 2023-10-02

**Authors:** Nicolle Kränkel, Ursula Rauch-Kroehnert

**Affiliations:** 1 Deutsches Herzzentrum der Charité, Klinik für Kardiologie, Angiologie und Intensivmedizin, Campus Benjamin-Franklin (CBF), 12203 Berlin, Germany; 2 DZHK (German Centre for Cardiovascular Research), Partner site Berlin, Berlin, Germany; 3 Friede Springer—Centre of Cardiovascular Prevention @ Charité, Charité—University Medicine Berlin, Berlin, Germany

**Keywords:** Drug development, Cell biology, Drug discovery

In their research article in *Nature* in March 2023, Fabio Zani and colleagues reported that high concentrations of the zero-calorie artificial sweetener sucralose affected the proliferation and differentiation of T cells toward interferon-γ-producing subsets, with no effect observed for B cells and myeloid cells.^1^ The publication has raised high hopes in the lay press on a widely available cure for patients suffering from autoimmune diseases.

**Fig. 1:**
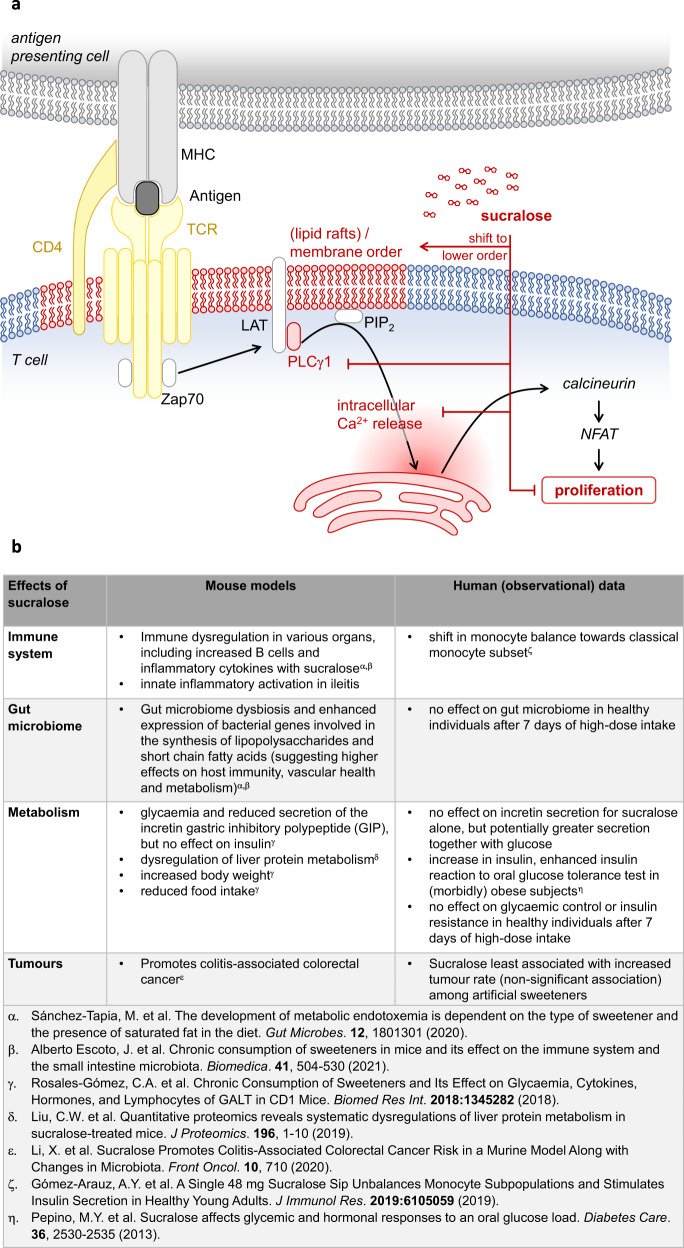
**a** Interference of high sucralose concentrations with TCR signaling, a summary of proposed mechanisms. High concentrations of sucralose are proposed to shift T-cell membrane order to a lower order, potentially associated with differences in protein allocation to lipid rafts. This might result in a greater spatial distance of PLC-γ1 from the (activated) T-cell receptor and consequently interrupt signal transmission. Intracellular Ca^2+^ release and T-cell proliferation, normally promoted by PLC in a PIP_2_-dependent manner, were impaired.^1^ Red arrows/outline indicates signaling events observed by Zani et al. Black/blue/yellow text and outline represent signaling events described previously. Pathways not described in the publication by Zani et al. (including PI3K/AKT, PI3K/PDK-1, Ras/MAPK/ERK, PKC-θ/NF-κB) are omitted here. AKT protein kinase B, CD4 cluster of differentiation 4, ERK extracellular signal-regulated kinase, LAT Linker for activation of T cells, MAPK mitogen-activated protein kinase, MHC major histocompatibility complex, NFAT nuclear factor of activated T cells, NF-κB nuclear factor kappa-light-chain-enhancer of activated B cells, TCR T-cell receptor, PDK-1 phosphoinositide-dependent kinase-1, PI3K phosphoinositide 3-kinase, PIP2 phosphatidylinositol-4,5-bisphosphate, PKC-θ protein kinase C theta, PLCγ1 phospholipase C gamma 1, Zap70 ζ-chain associated protein kinase of 70 kDa. **b** Comparison of sucralose effects reported in murine models and human observational studies and trials

Sucralose interfered with T-cell membrane order and limited PLCγ1-dependent induction of intracellular calcium release into the cytoplasm (Fig. 1a).^1^ Cell type-specific differences in cell membrane lipid composition are suggested by the authors as an explanation for the effect of high concentration sucralose, specifically in T cells. Indeed, lipid rafts compartmentalize signaling molecules in a spatially and temporally dynamic manner, thereby providing a means to selectively and flexibly permit or avoid activation of individual signaling cascades. This principle has been acknowledged to be crucial for T-cell expansion. Several mechanisms by which sucralose might potentially affect lipid raft formation and/or targeting of individual proteins to lipid rafts are conceivable, while keeping in mind the non-stable interaction of sucralose with T-cell membranes.^1^ More detailed molecular characterization of lipid rafts and functional interaction of proteins therein in the presence of sucralose, also in a concentration-dependent manner, may provide further insight into underlying molecular mechanisms.^1^ With respect to a potential clinical application in humans, it would be worthwhile to examine whether the membrane composition of T cells is indeed particularly sensitive to sucralose or whether other cell types are similarly affected by sucralose. This includes cells from the myeloid lineage, which are receptive to molecular patterns including glycated metabolites and cell components, and platelets which can be activated by another artificial sweetener, erythritol.^2^ Inter-individual variations in sucralose plasma levels might then translate into differential cell type-specific effects.

T-cell-mediated autoimmune responses and organ damage were reduced by high-dose sucralose in models of T-cell-induced colitis and type 1 diabetes mellitus.^1^ Despite the experimental nature of data with limited transferability to humans due to differences in immune system function, genetic variability between the species, and high heterogeneity in genetic background and lifestyle in humans (Fig. 1b), the potential benefit of high-dose sucralose on T-cell proliferation and effector function merits further studies for conditions of uncontrolled T-cell activation in humans. Notably, potential risks of high-dose sucrose ingestion might include a dampened T-cell-mediated anti-tumor response, as shown in sucralose-treated tumor-bearing mice and reduced defense against infections.^1^ Thus far, human data are mainly observational or from small cohorts and with lower dosage, providing no strong information on a sucralose effect on human immune response in clinically relevant settings. Albeit artificial sweeteners have been associated with different rates of cancers, the weakest associations were observed for sucralose.^4^

In contrast to several previous rodent models, no systemic effect of sucralose on the gut microbiome was seen in the current study. Ad libitum uptake of sucralose did not worsen morphological features of ileitis in a previous murine model of Crohn’s disease but did cause gut dysbiosis in the ileitis model and in the ileitis-free parental mouse strain.^3^ In the ileitis model, but not in the ileitis-free control group, sucralose was associated with increased myeloperoxidase activity—an indication of innate immune activation—and penetration of bacteria into gut tissues.^3^

Sucralose at dietary concentrations has been tested in numerous studies in humans and observational data—limited by mixed use of various artificial sweeteners—are available for large populations.^4^ Except for changes in the microbiota, consumption of sucralose in doses achievable by normal diet was not demonstrated to cause harmful effects in healthy humans (Fig. 1b). As opposed to the study situation, several artificial sweeteners are usually mixed in a person´s diet.^4^ Metabolic and immunologic effects of sucralose appear to differ if consumed alone or in combination with glucose, as well as in morbidly obese persons (Fig. 1b).^5^ Thus, combination with other food components in human diet as well as gut and metabolic health status may alter individual sucralose uptake and plasma levels and effects on immunity and metabolism.

Differences between experimental findings in the current study and previous pre-clinical and clinical reports might also be explained by the extremely high dose of sucralose used by Zani et al.^1^. The authors point out that a sucralose concentration of 1 µM is high, but achievable in humans by consumption of 250 mg sucralose, the maximum acceptable daily intake as defined by the European Food Safety Authority. Nevertheless, similar doses would usually not be reached by dietary consumption of artificially sweetened food or drinks, especially given the great sweetness of sucralose (ca. 600× higher than that of glucose). The long-term safety of artificial sweeteners is a recurring concern, with sucralose considered one of the safest.^4^ Long-term data on sucralose effects on human health when given daily at doses comparable to those used by Zani et al. are not available and will be needed prior to a potential clinical exploitation in patients with autoimmune diseases. Considering potential infection and tumor risk as well as practical issues of palatability, very likely a potential clinical application would constitute in a short-term application limiting acute autoimmune flares rather than a long-term preventive treatment. The observation that sucralose effects appeared to be reversible after washing-out of sucralose would improve the safety profile if replicated in humans on a systemic level.

In conclusion, randomized prospective clinical studies are warranted to determine whether the authors’ hypothesis that high doses of sucralose may be beneficial for various conditions arising from unrestrained T-cell activity in humans is more than a “sweet dream”.
